# Monitoring vegetation- and geodiversity with remote sensing and traits

**DOI:** 10.1098/rsta.2023.0058

**Published:** 2024-04-01

**Authors:** Angela Lausch, Peter Selsam, Marion Pause, Jan Bumberger

**Affiliations:** ^1^ Department of Computational Landscape Ecology, Helmholtz Centre for Environmental Research-UFZ, Permoserstr. 15, 04318 Leipzig, Germany; ^2^ Department of Physical Geography and Geoecology, Martin Luther University Halle-Wittenberg, Von-Seckendorff-Platz 4, 06120 Halle, Germany; ^3^ Department of Architecture, Facility Management and Geoinformation, Institute for Geoinformation and Surveying, Bauhausstraße 8, 06846 Dessau, Germany; ^4^ Department of Monitoring and Exploration Technologies, and; ^5^ Research Data Management—RDM, Helmholtz Centre for Environmental Research UFZ, Permoserstraße 15, 04318 Leipzig, Germany; ^6^ German Centre for Integrative Biodiversity Research (iDiv) Halle-Jena-Leipzig, Puschstraße 4, 04103 Leipzig, Germany

**Keywords:** remote sensing, traits, vegetation diversity, geodiversity, monitoring

## Abstract

Geodiversity has shaped and structured the Earth's surface at all spatio-temporal scales, not only through long-term processes but also through medium- and short-term processes. Geodiversity is, therefore, a key control and regulating variable in the overall development of landscapes and biodiversity. However, climate change and land use intensity are leading to major changes and disturbances in bio- and geodiversity. For sustainable ecosystem management, temporal, economically viable and standardized monitoring is needed to monitor and model the effects and changes in vegetation- and geodiversity. RS approaches have been used for this purpose for decades. However, to understand in detail how RS approaches capture vegetation- and geodiversity, the aim of this paper is to describe how five features of vegetation- and geodiversity are captured using RS technologies, namely: (i) trait diversity, (ii) phylogenetic/genese diversity, (iii) structural diversity, (iv) taxonomic diversity and (v) functional diversity. Trait diversity is essential for establishing the other four. Traits provide a crucial interface between *in situ*, close-range, aerial and space-based RS monitoring approaches. The trait approach allows complex data of different types and formats to be linked using the latest semantic data integration techniques, which will enable ecosystem integrity monitoring and modelling in the future.

This article is part of the Theo Murphy meeting issue ‘Geodiversity for science and society’.

## 1. Introduction

Climate change, land-use change, biological invasions, and the alteration and loss of natural bio- and geodiversity are causing rapid changes worldwide, from local to large scale [1]. There is therefore an urgent need for financially and temporally feasible working approaches for the qualitative and quantitative monitoring of biodiversity, geodiversity and their interactions. Since about 1985, remote sensing (RS) has introduced new and increasingly better methods for continuous ecosystem monitoring with the global monitoring of land cover by the Landsat mission, which quantitatively and qualitatively measures changes in vegetation diversity [2,3], geodiversity [4,5], soil properties [6], geomorphology [7] or hydrology [8], as well as the intensification and urbanization of landscapes [9,10]. Various RS technologies can be used to assess the status, changes and disturbances of characteristics, phylogenetics, structure, taxonomy as well as ecosystem functions, interactions and feedback mechanisms from the local to the global scale [11].

Recent technological developments and satellite missions such as the DLR's Earth Sensing Imaging Spectrometer (DESIS, [12]), the Hyperspectral Environmental Mapping and Analysis Program (EnMap, [13] or the first spaceborne GEDI Ecosystem Lidar [14]) are largely available free of charge to provide a deeper understanding of processes and accurate estimates of traits in vegetation and soil properties as a result of ecological pattern and their interactions. NASA's future Surface Biology and Geology (SBG) missions (https://sbg.jpl.nasa.gov/) with the Hyperspectral Infrared Imager (HyspIRI) will be particularly important for the RS-based monitoring of vegetation- and geodiversity [15].

The reason why RS can capture traits and trait variations of vegetation- and geodiversity is that the spectral reflectance and absorption of pixels in an optical RS image is the result of interactions between light (the atmosphere), the phylogenetic, biophysical, biochemical, morphological, physiological, phenotypic, structural, taxonomic, and functional traits of plants [16] and the traits of geodiversity [5,7], and the interactions between vegetation- and geodiversity [17].

The basis of the trait approach is the spectral variation hypothesis (SVH) approach [18]. The SVH assumes that the pixel-to-pixel variability of the spectral response in an RS image is determined by numerous factors. Environmental heterogeneity, the diversity of biochemical and structural traits of leaf and canopy properties, and functional vegetation properties and their responses through interactions with topography, soil and geodiversity all play a role [18,19]. As these characteristics are related to species diversity, among other things, spectral texture variations can be quantified as indicators of plant species diversity [20–22]. Thus, areas of high spectral heterogeneity in an RS image are areas of high species diversity and environmental heterogeneity, with a variety of ecological niches available, and therefore consisting of more species and habitats [18,23].

Traits are thus closely linked to the genotype/phenotype, structural, taxonomic and functional characteristics and processes of the ecosystem [24]. Traits and their variation are therefore filters of ecosystem condition, vitality, stress, processes, disturbance or resource limitation [25,26]. Furthermore, traits are a proxy for land use intensity and urbanization [9,10]. For example, land use intensity depending on its process characteristics (duration, consistency, extent, dominance, intensity or overlap), leads to characteristic spatio-temporal spectral responses in the RS image, which can be quantified by spectral indicators.

Thus, RS and the trait approach provide access to monitoring and indicator derivation of complex ecosystem properties, the genesis, structures and functions of vegetation- and geodiversity, and their interactions. To understand how RS technologies can monitor and quantify the five characteristics of vegetation- and geodiversity, the following objectives of this paper are as follows: (I) to understand the monitoring of traits-, phylogenetic-, structural-, taxonomic- and functional vegetation- and geodiversity using RS. (II) to demonstrate the link between *in situ* and RS approaches to monitoring vegetation- and geodiversity.

## 2. Monitoring vegetation diversity with RS and the trait approach

### (a) Definition and characteristics of vegetation diversity in the context of remote sensing

Vegetation diversity comprises the variety of plant species that occur in a given area or ecosystem and refers to the variability among plants, which includes both genetic differences within a species and the variety of different species. Vegetation diversity is defined by the following characteristics.

• Species richness: this refers to the number of different plant species in an ecosystem.
• Genetic diversity: there can be a large number of genetic variations within a species. This genetic diversity is crucial to the adaptability and survival of species.
• Structural diversity: this includes the physical forms and structures of plants, from grasses and shrubs to large trees. This diversity creates different habitats and is important for the ecosystem.
• Functional diversity: different plants perform different functions in an ecosystem, such as photosynthesis, providing food and habitat for animals, or cycling nutrients.
• Ecological diversity: this refers to the variety of ecosystems within a larger area, with each ecosystem supporting its own unique vegetation.
• Species evenness: this refers to how evenly individuals of a plant species are distributed compared to other species in an area.
• Seasonality and dynamics: vegetation diversity also takes into account temporal changes in the composition and abundance of plant species, influenced by seasons, climate change and other environmental factors.

However, in the context of monitoring vegetation diversity using RS, these characteristics are not useful as RS can capture traits and trait variation of plants, vegetation and communities. The spectral reflectance and absorption of pixels are the result of interactions between light (the atmosphere), phylogenetic/genetic, biophysical, biochemical, morphological, physiological, phenotypic, structural, taxonomic and functional traits of plants [16], as well as their interactions between vegetation- and geodiversity [17]. Therefore, in the context of monitoring vegetation diversity using RS, other traits are required, namely:

1) Plant trait diversity, which represents the diversity of chemical, biochemical, physiological, morphological, structural, textural or functional characteristics of plants, populations, communities that affect and interact with, and are influenced by phylogenetic-, taxonomic-, structural- and functional diversity.
2) The phylogenetic diversity of plants is the diversity of the length of evolutionary pathways associated with a particular set of phylogenetic, taxa, structures and functions of vegetation diversity. Therefore, groups of plant traits, taxa, structures and functions that maximize the accumulation of functional diversity of vegetation diversity are identified.
3) Structural diversity of vegetation is the diversity of composition and the configuration of structural features in plants, populations, communities, habitats and bioms.
4) Taxonomic diversity is the diversity of plants, which differ from each other from a taxonomic point of view.
5) The functional diversity is the diversity of functions and processes and their soil–water–atmosphere interactions and intra- and interspecific interplay in plants, populations and communities.

A clear separation and assignment of the five characteristics of vegetation diversity is not always possible, but nevertheless helps to monitor, assign and assess the various indicators of *in situ* and RS approaches, as well as to understand the links between both approaches.

### (b) Methods for monitoring vegetation diversity

There are two methods for monitoring vegetation diversity and the influences and changes in vegetation traits as a result of intensification, stress, disturbance and resource limitations. These are *in situ* or field measurements by biologists or ecologists, and the RS monitoring.

*In situ* observation refers to the direct recording, identification and monitoring of plant species, communities and habitats or landscapes by taxonomists and field ecologists. *In situ* approaches to characterize vegetation diversity were standardized early on and are based on different species concepts [27]. The most important of these are the phylogenetic species concept (PSC) [28], the biological species concept (BSC) [29] and the morphological species concept (MSC) [30].

**Figure 1.**
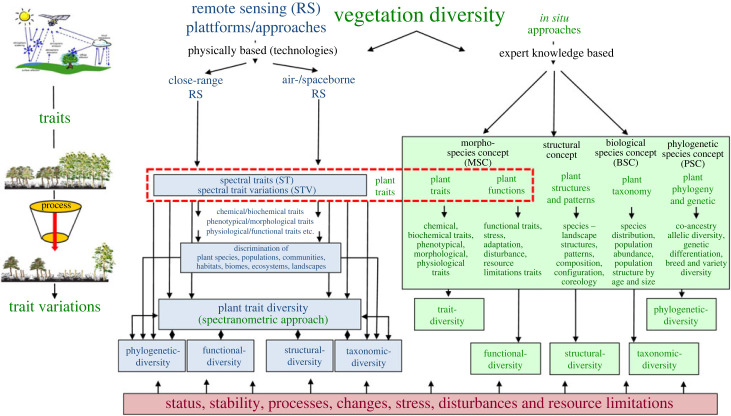
*In situ* and remote sensing approaches, common links between remote sensing and *in situ* for monitoring the five characteristics of vegetation diversity. Vegetation characteristics (traits) are the crucial link between *in situ* and RS monitoring approaches (modified after Lausch *et al*. [11]). (Online version in colour.)

Satellite imagery maps the Earth's surface on a grid with comparatively low/coarse spatial resolution, but unlike most other measurement techniques, the data are recorded in time and space. Repeated data acquisition allows the effects of seasonal cycles, developments over five decades and the movement of climatic zones to be recorded in time and space. RS technologies are mostly based solely on the spectral reflectance values of the remotely sensed terrestrial or aquatic surface. All RS sensors are non-contact with vegetation and are located at distances ranging from a few millimetres to thousands of kilometres. In addition, sensors are installed on platforms such as analytical spectral devices (ASD), cameras and sensors in a laboratory, wireless sensor networks (WSN), towers (short-range RS techniques), drones, aircraft (airborne RS) and satellites (space-based RS). RS captures biochemical, biophysical, physiognomic, morphological, structural, phenological and the functional traits of plants at all levels of organization, ranging from the molecular and individual level to communities and the whole ecosystem [31]. Optical RS monitoring is based on the principles of imaging spectroscopy across the electromagnetic spectrum from the visible to the microwave range [23]. Compared to *in situ* approaches and in particular the trait approach of the Morphological Species Concept (MSC) [30], RS approaches are not able to capture all traits and trait variations. The traits and trait variations that can be captured by RS sensors are therefore referred to as spectral traits (ST) or spectral trait variations (STV). Traits and trait variations are crucial for linking and bridging gaps between *in situ* and RS approaches to monitor and assess the changes, conditions, stress, disturbance or resource limitations of vegetation diversity and thus to assess vegetation health [11,32,33]. RS approaches are able to detect five different characteristics of vegetation diversity, namely: plant traits, phlyodiversity, functional diversity, structural- and taxonomic diversity (figure 1), which are described in more detail in the following chapter.

**Figure 2.**
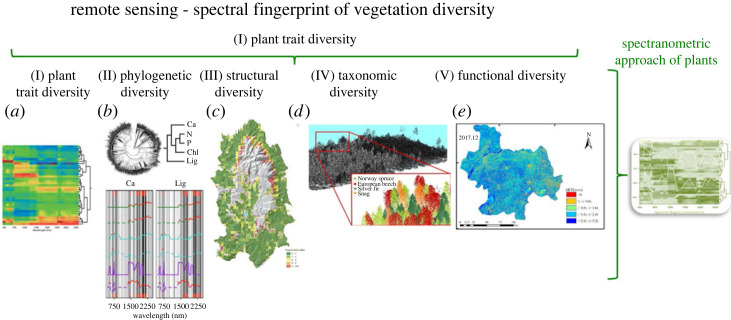
All five characteristics of vegetation diversity can be monitored using RS technologies. The individual characteristics of vegetation diversity are illustrated by means of examples, namely: (*a*) plant trait diversity: a spectroscopic cluster analysis of Kaua'i rainforest species based on their reflected light from 400 to 2500 nm. The colour codes show the spectral signatures of each species, with yellow–red and green–blue indicating high and low reflectance, respectively. The dendrogram on the right shows the spectranomic clustering of the species (Asner & Martin [34]). (*b*) Plant phylogenetic diversity: plant phylogenetic information results in specific chemical and biochemical traits in plants, that can be monitored with hyperspectral remote sensing technology. Mean reflectance spectra for each site (coloured lines) and range of all reflectance spectra for all sites included in the study (grey area); phylogenetic signal, as Pagel's lambda, of species reflectance coefficients at each site for the phylogenetic signal, as Pagel's lambda, of species reflectance coefficients at each site for four representative functional traits, superimposed on the multimodel ensemble regression coefficients for representative functional traits, superimposed on the multimodel ensemble regression coefficients (grey bars) as a measure of band importance for each trait model. Site-specific lambda spectra are shown (grey bars) as measures of band importance for each trait model (from McManus *et al*. [35]). (*c*) Structural diversity of vegetation: Bucegi Natural Park forest habitat fragmentation maps 2020 derived from Sentinel 2-MSI imagery (from Olariu *et al*. [36]). (*d*) Taxonomic diversity of vegetation: classification of tree species using triple-wavelength ALS in a temperate forest (from Amiri *et al*. [37]). (*e*) Functional diversity of vegetation: spatial distribution of evapotranspiration seasons in 2017 in the Lower Yangtze River based on Landsat 8 remote sensing data (from Song *et al*. [38]). (Online version in colour.)

All five characteristics of vegetation diversity can be monitored using RS technologies which are: (a) plant trait diversity, (b) phylogenetic diversity of plants, (c) structural diversity, (d) taxonomic diversity and (e) functional diversity. Based on these five characteristics, the spectral fingerprint of the vegetation should be mapped in the form of the spectranometric approach (figure 2).

### (c) Monitoring the trait diversity with RS

‘Plant trait diversity, which represents the diversity of chemical, biochemical, physiological, morphological, structural, textural, or functional characteristics of plants, populations, communities that affect and interact with, and are influenced by phylogenetic-, taxonomic-, structural- and functional diversity' (see ch. 2.1).

RS captures biochemical, biophysical, physiognomic, morphological, structural, phenological and functional traits of plants, ranging from the molecular, individual level to communities and the whole ecosystem [31,39]. Compared to *in situ* approaches, especially the trait approach of the Morpho Species Concept (MSC [30]), where plant traits are the focus, RS approaches are not able to capture all traits and trait variations of plants. However, the spectral plant traits that can be detected using RS data are the only and crucial methodological link between *in situ* and RS monitoring approaches to monitor and assess the changes, conditions, stress, health, disturbances or resource limitations of vegetation.

The first step in RS is to record plant traits and trait variation (figure 1), which allows the recording and quantification of plant trait diversity, from which Asner and Martin first defined the spectral approach [34]. Furthermore, spectral traits form the basis for plant discrimination using RS. Thus, RS can only discriminate plants from each other if the plants differ in their traits or trait variations. Spectral traits are also the crucial basis for the RS-based quantification of structural and functional vegetation diversity.

Spectral traits of plants are recorded by RS either as single traits, such as plant phenology, leaf carbon or nitrogen content, or they result from a combination of different spectral traits, such as ecological strategy types, biomass, vegetation structure or leaf area index. Spectral traits can be captured either by direct indicators (chlorophyll, xanthophyll or water content) and/or indirectly through interactions with geodiversity traits (evapotranspiration, soil moisture, plant strategy types) [40]. For example, heavy metals [34], plant diseases [41] or the application of pesticides [42] to crops lead to biochemical-biophysical molecular changes in plant traits that can be detected by RS technologies.

Satellite images are acquired at constant intervals. The frequency of image acquisition varies with the spatial resolution of the sensors. Daily images are acquired by the MODIS system with a rather coarse resolution of 250 m, or by the Landsat (USGS/NASA) or Sentinel 2 (ESA) systems with a resolution of 10–30 m and a repeat rate of 1–2 weeks. The Sentinel 1 system uses microwaves and has a repeat rate of 2–3 days with a resolution of 10 m. All these systems are available to the public and free of charge. In each case, the data collection is dense enough to follow seasonal changes, although the images are sometimes useless because clouds cover the Earth's surface. Most of the systems available to the public are developments from the past 10 years but optical and infrared image data provided by the Landsat system (USGS/NASA) are now available for the last 50 years. This makes it possible to monitor long-term trends and their spatial distribution for any point on the globe. Regular image acquisition allows statistical analyses that can reveal trends or local pecularities that are normally obscured by seasonal cycles. In addition, the long-term variability or stability of landscape parameters can be quantified if data from different decades can be compared.

Due to their high spectral resolution, hyperspectral satellites (DESIS, EnMAP, InSPIRI) are particularly suitable for detecting shifts, changes and disturbances in plant traits. Spectral traits and trait variations are filters and proxies for phylogenetic-, epigenetic-, land-use intensity or soil characteristics induced changes and disturbance processes triggered by different drivers and stress indicators.

### (d) Monitoring the phylogenetic diversity with RS

‘The phylogenetic diversity of plants is the diversity of the length of evolutionary pathways associated with a particular set of phylogenetic taxa, structures and functions of vegetation diversity. Therefore, groups of plant traits, taxa, structures and functions that maximize the accumulation of functional diversity of vegetation diversity are identified' (see ch. 2.1).

Phylogenetic diversity of vegetation not only promotes resilience and stability, but is also a key indicator of ecosystem functionality [43]. Therefore, the measurement of phylogenetic diversity is crucial. Hyperspectral RS satellites such as the Environmental Mapping and Analysis Program—EnMAP [44] or the forthcoming HyspIRI [45] with their high spectral resolution, are useful for the assessment of phylogenetic vegetation diversity because they can record a large number of different plant traits simultaneously. Asner & Martin [34] developed the first ‘spectranomics approach' based on imaging spectroscopy, which links phylogenetic, taxonomic and functional vegetation traits based on the phylogenetic traits of plants with the chemical phylogeny and material-structural and functional composition and development of plants [46]. Following this approach, 21 biochemical elements with their biochemical properties (e.g. content of different photosynthetic pigments, nitrogen, phosphorus, polyphenols, cellulose, lignin or water content in leaves) were recorded using hyperspectral RS data [34]. Thus, the spectranometric approach generates the spectral fingerprint of each plant species and the tree crown is generated according to its phylogenetic and chemical characteristics, which are based on the similarity and uniqueness of the chemical composition of plant taxa and communities [46]. Schweiger *et al*. [47] highlight that 97% of phylogenetic diversity in plants is based on an integrative spectral diversity indicator based on the leaf spectrum [24]. Cavender-Bares *et al*. [27] investigated the link of leaf spectrum using hyperspectral RS technology with genetic and phylogenetic variation in oaks and found spectral similarity to be significantly associated with phylogenetic similarity among oak species.

### (e) Monitoring the structural diversity with RS

‘Structural diversity of vegetation is the diversity of composition and the configuration of structural features in plants, populations, communities, habitats and bioms' (see ch. 2.1).

Natural and anthropogenic disturbances lead to changes in the structural diversity of vegetation. Thus, there is a direct relationship between the structural characteristics of plant traits in response to natural and human influences such as land use intensity. As structural traits occur at all levels of biological organization, structural vegetation diversity (composition and configuration) can be molecular, chemical and biochemical [48,49] at the phylogenetic level [35], at the organismal level [50] and species such as patterns of ecophysiological leaf traits and spectral response among life trees forms [51], at the population and community level [52] up to biomes, ecosystems and landscape types, all of which can be captured by RS [53]. Due to their high spectral resolution, hyperspectral RS techniques are particularly suitable for monitoring structural features compared to multispectral RS. Thus, heterogeneity [54], plant species diversity or richness of plant species [51], or fragmentation [55] can be recorded. These variables are also potentially suitable for monitoring and describing neighbourhood relationships, area, density, size and shape characteristics related to habitats, urbanization or land use intensity, as well as for assessing the degree of naturalness and homogenization of the vegetation. In this way, RS technologies can capture vegetation structures not only in two dimensions, but also in three diemensions with the first spaceborne 3D-GEDI lidar satellite [14,56]. The addition of radar RS technologies extends the range of structural plant characteristics that can be monitored.

### (f) Monitoring the taxonomic diversity with RS

‘Taxonomic diversity is the diversity of plants, which differ from each other from a taxonomic point of view' (see ch. 2.1).

Taxonomic diversity and the abundance of different plant species are key parameters for describing change, status, stability and resilience in ecosystems [57]. RS technologies, in especially hyperspectral imaging spectroscopy, are well suited to capture different traits and trait variation in plants. Nevertheless, RS-based detection of taxonomic vegetation diversity has its limitations. For example, different taxa in plant species, communities, vegetation types and biomes can only be distinguished using RS technologies if the plant taxa or communities differ from each other in traits and/or trait variation. Thus, different plant taxa can only be distinguished from each other if their species-dependent (phylogenetic) characteristics as well as their developmental processes (senescence or phenological characteristics) form traits (flowering characteristics, time of flowering and maturity, biochemical differences, growth forms etc.) that can be detected by means of RS. Thus, due to the high spectral resolution, a large number of different plant traits and trait variations can be detected using hyperspectral RS sensors. Compared to broadband RS techniques (multispectral RS sensors such as Landsat, Sentinel-2), hyperspectral RS sensors are very well suited for mapping tree species and tree communities [58], floral composition [59] or invasive plant species [60].

### (g) Monitoring the functional diversity with RS

‘The functional diversity is the diversity of functions and processes and their soil–water–atmosphere interactions and intra- and interspecific interplay in plants, populations and communities' (see ch. 2.1).

The trait approach (morphological species concept, MSC) to vegetation diversity is important to improve our understanding of why organisms live where they do, and how they respond or adapt to environmental change [61]. The trait approach is a proxy and indicator for the functionality of plants as well as trade-offs and ecosystem services [62,63]. As RS can capture traits and their variation, RS technologies are able to capture the state and change of functional vegetation diversity from local to global scales, which is one of the central tasks of RS. RS technologies and in particular hyperspectral sensors are very well suited for the detection and change of vegetation functions such as photosynthesis, carbon sequestration or evapotranspiration of plants [38].

Functional plant types (PFTs) are functional convergences caused by stress conditions or limited environmental resources that can be detected by the RS-based detection of plant traits [23]. The most important example of PFTs are the CSR strategy types of [64], where plant traits change according to the disturbance regimes and stressors affecting them. CSR strategy types are PFTs [64], where the composition of the community consists of ruderal plants (Ruderal-R), competitive plants (Competitor-C) and stress tolerant plants (Stresstolerator-S) [65]. Plant traits such as phenology, flowering onset, flowering time, canopy height, growth forms, specific leaf area and geometry are essential for determining and assigning plant species in the CSR trait space [66]. Furthermore, PFTs also play a crucial role in vegetation functions and in the establishment of plant functional types and complex plant strategy types to form a kind of vegetation adaptation [66,67].

## 3. Monitoring geodiversity with RS and the traits approach

### (a) Definition and characteristics of geodiversity in the context of remote sensing

Geodiversity refers to the variety of geological (rocks, minerals), geomorphological (landforms, topographic features) and pedological (soil types) features of a landscape or region. It encompasses the diversity of non-biological components of the Earth and its surface. This definition also includes the processes that have shaped these geological structures and forms over geological time. Characteristics of geodiversity are:

• Variability of rock types: geodiversity includes the variety of rock types and minerals that form the basis of the Earth's crust.
• Soil types and structures: different soil types, their composition and structure are also part of geodiversity.
• Landforms: this includes the variety of physical forms of the Earth's surface, such as mountains, valleys, rivers, lakes, coastlines and other topographic features.
• Geomorphological processes: the processes that lead to the formation and modification of landforms, such as erosion, sedimentation, tectonic activity and volcanic events, are essential aspects of geodiversity.
• Geological aspects: geodiversity also includes an understanding of the Earth's history as recorded in geological strata, fossils and other geological features.
• Hydrogeological features: water-related geological features such as aquifers, springs and river systems are also part of geodiversity.

Geodiversity is important not only for understanding the Earth's history and geological processes, but also for nature conservation, as it provides the basis for many ecosystems and their biodiversity. It also plays an essential role in providing natural resources and shaping the landscape, which in turn influences cultural and aesthetic values.

However, in the context of monitoring geodiversity using RS, the above characteristics are not useful as RS can capture geodiversity traits and trait variation. The spectral reflectance and absorption of pixels is therefore the result of interactions between light (the atmosphere), mineralogical, bio/geochemical, bio/geo-optical, chemical, physical, morphological, structural, textural or functional characteristics of geodiversity and their interactions with vegetation. Therefore, in the context of monitoring geodiversity using RS, other characteristics are required, namely:

Geodiversity, can be described by its five characteristics, namely: (a) geomorphic trait diversity, (b) geomorphic genesis diversity, (c) geomorphic structural diversity, (d) geomorphic taxonomic diversity and (e) geomorphic functional diversity [7]. These five characteristics of geomorphodiversity exist on all spatial, temporal and directional scales of geomorphic organization and interact and influence each other, as well as affecting biodiversity and further spheres of geodiversity such as the lithosphere, hydrosphere or atmosphere, either directly or indirectly on all these scales. The five characteristics of geodiversity and geomorphodiversity are defined by Lausch *et al*. [7] as:

1) Geotrait diversity, which represents the diversity of mineralogical, bio-geochemical, bio-/geo-optical, chemical, physical, morphological, structural, textural or functional characteristics of geo components that affect, interact with, or are influenced by geogenese diversity, geotaxonomic diversity, geostructural diversity and geofunctional diversity.
2) The geogenese diversity is the diversity of the length of evolutionary pathways associated with a particular set of geotraits, geotaxa, geostructures and geofunctions of geodiversity. Therefore, groups of geotraits, taxa, structures and functions that maximize the accumulation of functional diversity of geodiversity are identified.
3) The geostructural diversity is the diversity of composition and the configuration geostructural features of geodiversity.
4) The geotaxonomic diversity is the diversity of its components, which differ from each other from a taxonomic point of view.
5) The geofunctional diversity is the diversity of geofunctions and processes and their intra- and interspecific interactions.

A clear separation and assignment of the five characteristics of geodiversity is not always possible, but nevertheless helps to monitor, assign and assess the various indicators of *in situ* and RS approaches, as well as to understand the links between both approaches.

### (b) Methods for monitoring geodiversity

Geodiversity is defined as the diversity of mineralogical, bio/geochemical, bio/geo-optical, chemical, physical, morphological, structural, textural and functional characteristics of soils, geomorphology, hydrology and the atmosphere. Important components in the monitoring of geodiversity are geo-trait diversity, geo-gene diversity, geo-taxonomic diversity, geo-structural diversity and geo-functional diversity [7]. Geodiversity is also studied using two types of monitoring approaches, the *in situ* approach and the RS approach.

Historically, scientists such as Humboldt [68] have developed and used numerous *in situ* measurement techniques to detect, record, characterize and monitor geodiversity features in order to assess anthropogenic and natural changes and disturbances and their impacts. Considerable knowledge about geodiversity has been gained through many years of practical experience and integration of the latest technologies in various fields such as fieldwork, laboratory experiments, microstructural investigations, analytical modelling, seismic investigations or geoelectrical investigations [69,70]. Fieldwork is essential to record genesis, structures, patterns and functions and understand processes, changes and disturbances of geodiversity. It also provides the basis for the calibration and validation of RS-based indicators and for data-driven modelling and prediction.

In RS-based monitoring, sensors detect geodiversity traits and trait variations [6]. However, a successful RS-based detection of geodiversity traits depends on the characteristics of the RS sensors and the spatio-temporal distribution (composition and configuration) of the geodiversity traits [7]. Only when RS technologies such as radiometric, geometric, spectral, angular or temporal resolution of RS sensors are specific to the detection of geodiversity traits and trait variations can they be detected with RS. For example, RS data can distinguish geodiversity traits when they differ in their characteristics, such as: different minerals such as carbonates, sulphates, chlorides, silicates, oxides, material types such as sand, rock, gravel, material properties such as shape, texture, colour or shape features such as curvatures of river loops, characteristics of river valleys, fracture steps, pits or slopes [7]. The detectability of geodiversity traits using RS technologies is therefore essential for the detection, differentiation, classification and monitoring of the five characteristics of geodiversity (geotrait diversity, geogene trait diversity, geostructural, geotaxonomic and geofunctional geodiversity).

In regions without vegetation cover, the detection of geodiversity using RS techniques is possible through direct RS indicators. The spectral RS signal is the result or integral of the state and changes, shifts and/or disturbances of geodiversity features. In regions covered by vegetation, water or ice, indirect indicators that are integral to the interactions and responses of geotraits to bacteria, algae, plants, populations, communities or landform features and their interactions can be used in addition to direct RS indicators [6,7,40].

**Figure 3.**
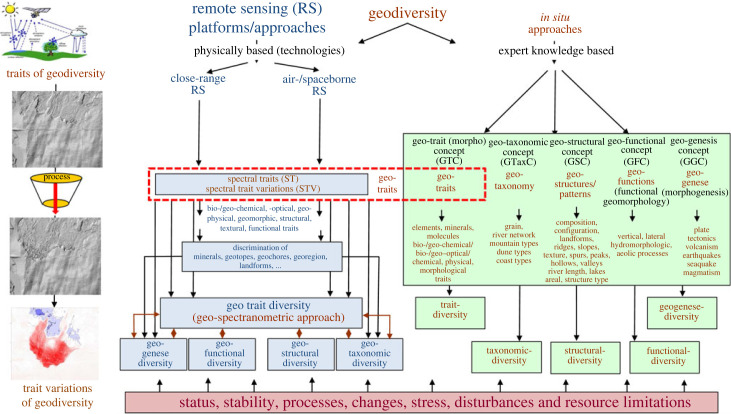
*In situ* and remote sensing approaches, common links between remote sensing and *in situ* for monitoring the five characteristics of geodiversity. The geotraits of geodiversity are the important link between *in situ* and RS monitoring approaches (modified from Lausch *et al*. [7]). (Online version in colour.)

Since about 1985, RS has provided new and successful technologies for the continuous, harmonized and efficient monitoring of geodiversity [4], geomorphodiversity [71], geohydrology [72], hydrology [73] and atmosphere, ranging from the local to the global scale. NASA's future SBG missions (https://sbg.jpl.nasa.gov/) with the HyspIRI are particularly useful for RS-based monitoring of geodiversity [15]. The HyspIRI satellite can be used to derive numerous RS-based indicators of vegetation and geodiversity such as plant physiology, functional traits and health, agriculture, natural habitats, urban development or water use and quality, inland and coastal aquatic ecosystems, physiology, as well as snow and ice accumulation, active surface changes or the impact of changing land use on the surface, energy, water, and C fluxes both continuously and globally (https://hyspiri.jpl.nasa.gov/). In the following chapters, we will explain in detail how RS can capture the five features of geodiversity, namely: geodiversity feature diversity, geodiversity geogenese, geodiversity structural diversity, geodiversity taxonomic diversity and geodiversity functional diversity (figure 3).

**Figure 4.**
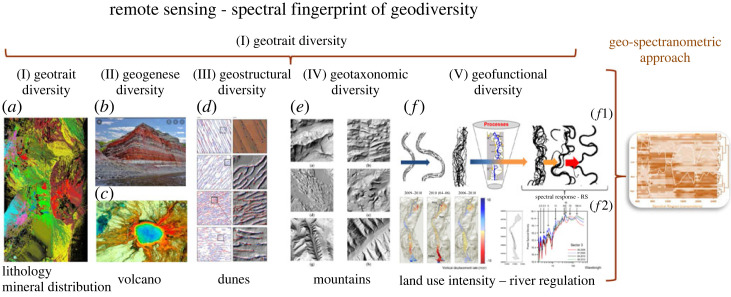
All five characteristics of geodiversity can be monitored using RS technologies. The individual characteristics of geodiversity are illustrated by means of examples, namely: (I) geotrait diversity, (*a*) AVIRIS hyperspectral RS data were used to classify mineral distribution and the geotraits in the Cuprite area, Nevada (from Clark *et al*. [74]); (II) geogenese diversity, (*b*) photo of the characteristic relief forms created by the exogenous and endogenous geogenese processes, (*c*) TIR image of part of the Siberian Trap supervolcano; (III) geostructural diversity, (*d*) derivation of dune pattern mapping with RS (from Shumack *et al*. [75]); (IV) geotaxonomic diversity, (*e*) classification of different mountain types using RS (from Farmakis-Serebryakova *et al*. [76]); (V) geofunctional diversity, (*f*1) processes of geogenese and river degradation lead to changes in morphometric river features, (*f*2) the morphometric changes can be recorded using RS data (reprinted with permission from Ventura *et al*. [77], Elsevier license number: 4856041399548). (*g*) The integration and combination of all five features form the basis of the geo-spectranometric approach and lead to the spectral fingerprint of geomorphology and geodiversity. All features and individual figures are explained in detail in the following chapters (from Lausch *et al*. [7]). (Online version in colour.)

All five characteristics of geodiversity/geomorphodiversity can be monitored using RS technologies. The individual characteristics of geodiversity are illustrated by means of examples, namely: (a) geotrait diversity, (b) geogenese diversity, (c) geostructural diversity, (d) geotaxonomic diversity and (e) geofunctional diversity. Based on these five characteristics, the spectral fingerprint of the geodiversity should be mapped in the form of the geo spectranometric approach (figure 4).

### (c) Monitoring the geotrait diversity with RS

‘Geotrait diversity, which represents the diversity of mineralogical, bio-geochemical, bio-/geo-optical, chemical, physical, morphological, structural, textural or functional characteristics of geo components that affect, interact with, or are influenced by geogenese diversity, geotaxonomic diversity, geostructural diversity and geofunctional diversity' (see ch. 3.1, from Lausch *et al*. [7]).

RS captures traits and trait variations of geodiversity and geomorphology ([6,7], figure 4.). RS can thus capture, monitor and compare the trait diversity of geodiversity on the basis of spectral traits (spectral indicators) and their changes. If the spectral traits of the geodiversity to be recorded differ in terms of their traits such as mineralogical, bio/geochemical, bio/geo-optical, physical, morphological (e.g. minerals such as silicates, oxides, carbonates, sulphates, chlorides and material types, such as sand, rock, gravel, soil types), material properties (texture, colours, shapes), structural, textural form features (i.e. river valleys, pits, slope gradients or river meanders), taxonomic units (water body types, mountain types, river network types) or functional characteristics (functional connectivity, hydrological dynamic indicators, slope stability) of their components, they cannot be distinguished from each other or recorded using RS technologies [7]. Recognition and differentiation of the traits in geodiversity is therefore a crucial basis for the monitoring of all geodiversity characteristics. In contrast to vegetation diversity, changes in geodiversity are subject to different temporal variations (short-term—extreme events, centuries or millennia), which means that disturbances or ongoing geoprocesses are often difficult or impossible to detect using RS.

A further distinction is made between direct and indirect RS-based methods for monitoring geodiversity. In regions without vegetation cover, geodiversity traits can be recorded by deriving direct RS indicators or traits (e.g. mineralogical composition, soil types, soil properties such as soil moisture, C-org. etc.). The spectral RS signal is the result or integral of the state and changes, shifts and/or perturbations of traits of geodiversity traits. In regions covered by vegetation, water or ice, in addition to direct RS indicators, indirect indicators may be used that are integral to the response of traits of bacteria, algae, plants, populations, communities or landforms and their interactions.

Monitoring the trait diversity of geodiversity is an integral part of geotrait, geogenesis, geostructural, geotaxonomic and geounctional geodiversity, whereby detection will depend on the RS characteristics (geometric, spectral, temporal, radial resolution) as well as on the spatio-temporal distribution and thus how well the traits of geodiversity can be detected by the RS technology available. Hyperspectral imagery can provide the spectral fingerprint (geospectral approach, figure 4) for local to global areas [7].

### (d) Monitoring the geogenese diversity with RS

‘The geogenese diversity is the diversity of the length of evolutionary pathways associated with a particular set of geotraits, geotaxa, geostructures and geofunctions of geodiversity. Therefore, groups of geotraits, taxa, structures and functions that maximize the accumulation of functional diversity of geodiversity are identified' (see ch. 3.1, from Lausch *et al*. [7]).

RS has added a new dimension to the monitoring of geogenese diversity, its characteristics, impacts, disturbances and biodiversity. RS can identify, monitor and describe geogene features (minerals, rock types), taxa (mountain species), structures (genesis patterns, lineaments) and functions (flow behaviour) that represent the tectonic architecture and its characteristics [78]. Detailed structure and pattern analyses using RS technologies help to interpret, classify, distinguish and thus identify the origin of different structures and patterns in the Siberian and Deccan Traps [78].

Therefore, RS-based lineament analyses are key elements in the interpretation of local, regional and continental geogenese structures [79]. Any naturally formed linear feature on the Earth's surface that is connected by processes of extension, compression, or strike-slip or that results from magmatic or metamorphic activity is called a lineament [80]. There are several geo-taxonomic forms of lineaments, including rock types, linear dolines, fault traps, fold hinges, faults, shear zones, dykes, mineralized veins, uplifted topography or contacts between elongated fractures or fault-related elongated valleys [79,81].

In addition to lineaments, terrain patterns or fluvial drainage patterns also provide important clues to the causes, trends and nature of subsurface structures that cannot be detected by RS [79]. Drainage patterns in flat terrain are usually dendritic; however, for a dome or rock structure, drainage patterns are radial and concentric [81]. Orthogonal, barbed and double drainages or compressed meanders are other examples of drainage patterns that control the flow of water through their structure [79,80,82].

### (e) Monitoring the geostructural diversity with RS

‘The geostructural diversity is the diversity of composition and the configuration geostructural features of geodiversity' (see ch. 3.1, from Lausch *et al*. [7]).

Thus, endogenous and exogenous processes are responsible for the formation of relief and form and have either led or are leading to the formation, structuring and modification of our Earth's surface and its ecosystems. Geomorphometry, structures, patterns, diversity, relief and topography are therefore critical to the functionality, feedback and resilience of geo- and biodiversity that influence Earth's surface processes and landforms [83]. Structures, patterns and sculptural forms are thus indicators of geodiversity processes and functions, providing not only crucial information about the nature and origin of the process, but also important clues about the direction and course of change through the characteristics of the process (length, consistency, extent, dominance, intensity or overlap). Spatio-temporal forms, structures and patterns of geodiversity also describe the degree of naturalness or anthropogenic influence (hemeroby) [84,85] on the ecosystem.

For example, land use intensity (LUI), urbanization, open-cast mining activities, intensified forestry practices or river regulation, alter evolutionary geomorphic structures and form patterns in some cases to such an extent that the original natural structures are difficult to capture. Numerous examples of geomorphic impact define the terrain of our present-day cultural landscape today, such as roads, buildings, towns, terraces, boundary developments, fallow land, ditches, canals or reservoirs. The characteristics of geostructures are therefore important footprints of human influence [10]. Important conclusions can be drawn about the functionality and resilience of the ecosystem. For example, the straightening of rivers leads to measurable morphometric changes in riverine landscapes that affect their functionality [86].

Structural diversity exists at all levels of geodiversity organization [7]. Therefore, structural features should be captured with different RS platforms at all spatio-temporal and directional scales of geodiversity. For the successful acquisition of geomorphological structure, such as topography, the sensor technologies and consequently the RS features should be chosen wisely. By capturing the detailed terrain structure of coastal regions using airborne lidar data, it has been shown that more than three times as many people are at risk from climate change and sea-level rise than previously calculated using less detailed Shuttle Radar Topography (SRTM)-DEM-RS data [87]. Thus, the RS technology used will also determine the quality of the model and the model prediction of landscapes change and disturbance. Ecological and hydrological model predictions are therefore only as good as the quality of the RS-based input data obtained [71].

Furthermore, structures and patterns are crucial for the differentiation of geodiversity taxa and thus for the characterization of taxonomic diversity, which is important with the help of RS (see ch. 3.4).

### (f) Monitoring the geotaxonomic diversity with RS

‘The geotaxonomic diversity is the diversity of its components, which differ from each other from a taxonomic point of view' (see ch. 3.1, from Lausch *et al*. [7]).

Processes of exogenous and endogenous geogenese, such as plate tectonics, mountain development or volcanism, have led to the formation of numerous geomorphological taxa (also known as types, classes or units), such as mountains, ranges, reliefs, volcanoes, channels, rocks, landforms, water types, river networks, dunes etc.), with specific geochemical, mineralogical and structural traits, forms or classes of forms. This taxonomic diversity, heterogeneity and richness of different geomorphic types determines the state, stability and resilience of the entire geosphere and biosphere, as they give rise to a wide variety of ecosystem processes, functions, forms and structural types, ultimately creating ecological niches. For example, the production of volcanic lava, gases or solid shapes different characteristic volcanic forms. The properties of the resulting volcanic products will also vary, i.e. they may be gaseous, viscous, have low viscosity or be solid. Cinder cone volcanoes, for example, were formed from loose material and have a characteristic conical shape with an inclination of 30–40˚, which led to the formation of the characteristic concave slope shape. The volcanic ash also created the vast grassy savannah areas of the Serengeti, preventing the encroachment and development of forest communities.

However, anthropogenic changes such as land use intensity, agricultural expansion, urbanization, climate change or resource extraction have influenced and shaped a variety of landforms and geomorphic types over thousands of years [88]. This has led to changes and the formation of distinct anthropogenic geomorphic types with strong anthropogenic features such as reservoirs, dams, canals, mines, terraces or roads, buildings and towns [10]. Anthropogenic geomorphic features such as linear structures, river straightening and the characteristic structures of terraces or mines can now be used to monitor the degree of human influence and improve the discrimination and classification of geomorphic types.

RS techniques can be used to record geodiversity traits [71], soil properties [6] or the interactions between above- and below-ground diversity [17] as well as biodiversity [89,90]. Different geomorphic taxa are distinguished by their different geomorphic traits, which are discriminated from each another using RS data. Discrimination in turn depends on the RS characteristics (spatial, spectral, temporal, radial resolution). Many RS technologies are used to detect human impacts and changes in geotaxa through LUI by using spectral image analysis, such as monitoring river degradation, terrain shaping [10] and changes to coastal structures using lidar [87] or urbanization (cities and roads) using multispectral, lidar or radar technologies [10].

### (g) Monitoring the geofunctional diversity with RS

‘The geofunctional diversity is the diversity of geofunctions and processes and their intra- and interspecific interactions' (see ch. 3.1, from Lausch *et al*. [7]).

Anthropogenic influences such as land use intensity, urbanization and river straightening have increasingly caused irreversible changes and disturbances to natural geomorphology from the nineteenth to the twenty-first century, resulting in significant disruptions to ecosystem functionality and resilience [10]. Using river straightening as an example, we briefly discuss the basic reasons why RS can capture the genesis as well as the structural and functional changes and processes.

During geogenese, divergent and convergent flow movements formed river meanders transverse to the general direction of the flow. River meanders are an expression of a stable, dynamic equilibrium between the river and the riverbed, leading to the formation of a characteristic fluvial biodiversity with a high self-purification potential. The geometry of meanders, both cut-off meanders and oxbow lakes, can vary greatly, as meanders are subject to constant changes in position. In the nineteenth century, the Upper Rhine (Germany) underwent measures for flood protection (reduction of flood-prone areas), low water regulation and hydropower development. The morphological effects of these measures on the Rhine changed the erosion and sedimentation behaviour of the river to such an extent that the flow velocity increased at the same time. This led to a strong vertical erosion of up to 7 m in the Rhine. The eroded material often resulted in the formation of sand and gravel banks, which caused the barrages to act as sediment traps, requiring further low water regulation measures [91]. River regulation or barrages thus lead to changes in the structural and functional characteristics of rivers, which in turn lead to variations in river characteristics and consequently aquatic biodiversity. The structural geomorphological changes of the original meanders or sediment displacements can now be detected with RS approaches, as these changes in fluvial characteristics lead to spectral responses in the RS signal (figure 4). Figure 4*f*2 shows an example of monitoring temporal changes in fluvial features—the vertical displacement rate of the river system from 2006 to 2010, using RS technologies (lidar). In addition to structural changes, hyperspectral technologies (HySPEX, AISA, CHIME or EnMAP) can also be used to detect changes in vegetation diversity and water quality [92] (increasing eutrophication, chlorophyll content and turbidity).

RS also plays a crucial role in monitoring topography and relief (DEM, DSM), from which a whole range of indicators can be derived that are essential variables for ecological models.

## 4. Conclusion and further research

Geodiversity has shaped and structured the Earth's surface through long-term as well as medium- and short-term processes at all spatio-temporal scales. Geodiversity is therefore the key controlling and regulating variable for the overall development and change to landscapes and biodiversity. The evolving changes, disturbances and interactions between geodiversity and vegetation diversity are complex, multidimensional and multi-scale in space, time, processes and drivers. Therefore, only standardized monitoring at all spatio-temporal scales of geodiversity and vegetation diversity is feasible in a cost-effective and timely manner. RS approaches have been used successfully for many years to achieve these goals.

RS captures traits and trait variation in vegetation and geodiversity. Furthermore, traits are the crucial interface between *in situ*, short-range, airborne and space-based RS monitoring approaches. As traits can be viewed as filters or proxies for monitoring status, change, disturbance or resource limitation, RS approaches allow the detection of these components. Similarly, traits exist at all spatio-temporal scales, allowing standardized monitoring using RS possible at all scales.

However, in order to understand how RS captures geodiversity and vegetation diversity, the aim of this paper is to describe in detail the monitoring of five characteristics of vegetation diversity and geodiversity with RS technologies, namely: (i) trait diversity, (ii) phylogenetic/geogenese diversity, (iii) structural diversity, (iv) taxonomic diversity and (v) functional diversity, with trait diversity forming the fundamental basis for the assessment of the other four characteristics. Similar to the approach of Diaz [78] (The global spectrum of plant forms and functions), ‘a global spectrum of geodiversity' based on traits, forms and functions of geodiversity could be created in the future using RS.

The monitoring of vegetation and geodiversity and their interactions is complex. Therefore, future monitoring requires a holistic and interdisciplinary approach and analysis tools that allow the coupling of *in situ* data, RS platforms, databases, and the derivation of spectral indicators and the integration of ecological models. The trait approach also allows the coupling of complex data with different data types and formats using the latest semantic data integration techniques, enabling monitoring and modelling of ecosystem integrity (see also https://research.csiro.au/ereefs/).

Furthermore, based on traits and remote sensing data, a freely available tool (ESIS- Ecosystem Integrity Remote Sensing/Modelling Tool and Service) was developed, which can help research, application and planning to better classify and model remote sensing data based on the trait concept in order to achieve an improved ecosystem and integrative process understanding. The tool is constantly being further developed and can be downloaded from GitLab (https://doi.org/10.5281/zenodo.8116370) [93].

## Data Availability

This article has no additional data.
